# Evaluation of the Enantiomer Specific Biokinetics and Radiation Doses of [^18^F]Fluspidine—A New Tracer in Clinical Translation for Imaging of σ_1_ Receptors

**DOI:** 10.3390/molecules21091164

**Published:** 2016-09-01

**Authors:** Mathias Kranz, Bernhard Sattler, Nathanael Wüst, Winnie Deuther-Conrad, Marianne Patt, Philipp M. Meyer, Steffen Fischer, Cornelius K. Donat, Bernhard Wünsch, Swen Hesse, Jörg Steinbach, Peter Brust, Osama Sabri

**Affiliations:** 1 Helmholtz-Zentrum Dresden-Rossendorf, Institute of Radiopharmaceutical Cancer Research, Department of Neuroradiopharmaceuticals, Leipzig 04318, Germany; m.kranz@hzdr.de (M.K.); w.deuther-conrad@hzdr.de (W.D.-C.); s.fischer@hzdr.de (S.F.); c.donat@imperial.ac.uk (C.K.D.); j.steinbach@hzdr.de (J.S.); 2 Department of Nuclear Medicine, University Hospital Leipzig, Leipzig 04103, Germany; Bernhard.Sattler@medizin.uni-leipzig.de (B.S.); Nathanael.Wuest@gmx.de (N.W.); Marianne.Patt@medizin.uni-leipzig.de (M.P.); Philipp.Meyer@medizin.uni-leipzig.de (P.M.M.); Swen.Hesse@medizin.uni-leipzig.de (S.H); Osama.Sabri@medizin.uni-leipzig.de (O.S.); 3 Division of Brain Sciences, Department of Medicine, Hammersmith Hospital Campus, Imperial College London, London SW7 2AZ, UK; 4 Pharmaceutical and Medicinal Chemistry, University Münster, Münster 48149, Germany; wuensch@uni-muenster.de; 5 Integrated Research and Treatment Center (IFB) Adiposity Diseases, University Hospital Leipzig, Leipzig 04103, Germany

**Keywords:** image based internal dosimetry, [^18^F]fluspidine, preclinical hybrid PET/MRI, radiation safety, σ_1_ receptors

## Abstract

The enantiomers of [^18^F]fluspidine, recently developed for imaging of σ_1_ receptors, possess distinct pharmacokinetics facilitating their use in different clinical settings. To support their translational potential, we estimated the human radiation dose of (*S*)-(−)-[^18^F]fluspidine and (*R*)-(+)-[^18^F]fluspidine from ex vivo biodistribution and PET/MRI data in mice after extrapolation to the human scale. In addition, we validated the preclinical results by performing a first-in-human PET/CT study using (*S*)-(−)-[^18^F]fluspidine. Based on the respective time-activity curves, we calculated using OLINDA the particular organ doses (ODs) and effective doses (EDs). The ED values of (*S*)-(−)-[^18^F]fluspidine and (*R*)-(+)-[^18^F]fluspidine differed significantly with image-derived values obtained in mice with 12.9 μSv/MBq and 14.0 μSv/MBq (*p* < 0.025), respectively. A comparable ratio was estimated from the biodistribution data. In the human study, the ED of (*S*)-(−)-[^18^F]fluspidine was calculated as 21.0 μSv/MBq. Altogether, the ED values for both [^18^F]fluspidine enantiomers determined from the preclinical studies are comparable with other ^18^F-labeled PET imaging agents. In addition, the first-in-human study confirmed that the radiation risk of (*S*)-(−)-[^18^F]fluspidine imaging is within acceptable limits. However, as already shown for other PET tracers, the actual ED of (*S*)-(−)-[^18^F]fluspidine in humans was underestimated by preclinical imaging which needs to be considered in other first-in-human studies.

## 1. Introduction

The existence of various tissues of the sigma opioid receptor (σ) was postulated first by Martin et al. in 1976 [1]; nowadays it has been proven to be a non-opioid receptor (Sigma Non-Opioid Intracellular Receptor 1; σ_1_ receptor). This receptor plays an important role in the cellular functions associated with the endocrine, immune, and nervous systems; however, the physiological function of the σ_1_ receptor is not yet fully understood [2]. Furthermore, this protein interacts with a variety of psychotomimetic drugs, including cocaine and amphetamines. Various diseases like neuropsychiatric and vascular diseases as well as cancer seem to be related to dysfunctions of the σ_1_ receptor [3,4,5]. Therefore, studying this protein with positron emission tomography (PET) could contribute to a better understanding and further evaluation of the pathophysiological role of σ_1_ receptors in diseases [6]. For imaging of σ_1_ receptors several radioligands were developed and used in human such as [^18^F]FPS [7] and [^18^F]FM-SA4503 [6]. The latter study showed that the σ_1_ receptor density is decreased in different brain structures in patients with early Alzheimer’s and Parkinson’s disease. Recently, our group developed and tested the chiral σ_1_ receptor ligand [^18^F]fluspidine in preclinical studies in mice and piglets [8], which revealed high brain uptake of the two enantiomers (*R*)-(+)-[^18^F]fluspidine and (*S*)-(−)-[^18^F]fluspidine along with marked enantioselectivity with regard to their biokinetics. As a consequence, the binding potential (BPnd) of (*R*)-(+)-[^18^F]fluspidine is 5- to 10-fold higher in comparison to (*S*)-(−)-[^18^F]fluspidine in σ_1_-rich areas of the porcine brain [9], most probably due to differences in their affinity towards σ_1_ receptors ((*R*)-(+)-[^18^F]fluspidine: *K*_i_ = 0.57 nM; (*S*)-(−)-[^18^F]fluspidine: *K*_i_ = 2.3 nM; [10]). These preclinical data indicated a suitability of both enantiomers of [^18^F]fluspidine for different clinical issues. For the first-in-human investigation of σ_1_ receptors in brain we have chosen (*S*)-(−)-[^18^F]fluspidine as the enantiomer with the faster pharmacokinetics for reasons of feasibility in clinical routine (German clinical trial register ID: DRKS00008321).

A radiation dose assessment, i.e., calculations of the absorbed and effective doses per unit activity administered is mandatory for the translation of novel radiotracers from preclinical to clinical study phases. These calculations are mainly based on biokinetic models using data obtained in biodistribution or imaging studies in animals. Usually rodents [11,12,13,14,15] or monkeys [16,17,18,19] are used and require the application of computational phantoms [20,21,22,23]. With rodents, both the organ harvesting method and the dynamic hybrid imaging method are feasible to collect biokinetic data which is later extrapolated to human anatomy (concerning organ mass and time scaling) [20]. By the organ harvesting method, the tissue activity concentration is quantified by gamma-counting and converted into percent of injected activity accumulated per organ (%ID) after dissection of the animals at different points post injection of a radiotracer. With the imaging method, the biokinetics of the radiotracer is investigated using clinical or small-animal PET/CT or PET/MRI systems. The activity in the organs as well as the weight is extracted after delineation with the help of the anatomical CT or MR images, and the organ-specific %ID values are calculated. Eventually, interspecies extrapolation of the respective animal data has to be performed to calculate the human effective dose. However, the standard procedure of these established models may lead to underestimation of radiation risk in humans as we could recently show with (−)-[^18^F]flubatine [24] and (+)-[^18^F]flubatine [25]. The preclinical dosimetry in mice revealed an underestimation of the effective dose in humans of up to 50% which could be improved only slightly when using piglets as larger species (underestimation ~38%).

In this work, we report on the dosimetry and biodistribution of both enantiomers of the σ_1_ receptor ligand [^18^F]fluspidine based on in vivo and ex vivo data from mice which we obtained by the dynamic hybrid PET/MR imaging method as well as by an organ harvesting study. Subsequently, we report on the first-in-human internal dosimetry using (*S*)-(−)-[^18^F]fluspidine obtained in four healthy volunteers. This direct comparison of preclinical with clinical data is assumed to advance the use of small animal PET/MRI for the assessment of the radiation risk of novel PET imaging agents in humans. The preclinical dosimetry reveals the ED for (*S*)-(−)-[^18^F]fluspidine and (*R*)-(+)-[^18^F]fluspidine comparable with other ^18^F-labeled PET imaging agents, despite significant differences of the EDs due to enantiomer specific tracer kinetics. The ED estimate from the first-in-human study confirmed that the radiation risk of (*S*)-(−)-[^18^F]fluspidine imaging is within accepted limits. However, as shown in previous studies, the ED in humans is underestimated by up to 50% by using preclinical imaging for internal dosimetry. This fact needs to be considered when applying for first-in-human studies based on preclinical biokinetic data scaled to human anatomy.

## 2. Results

In this study, we have investigated the preclinical dosimetry of both enantiomers of the σ_1_ receptor ligand [^18^F]fluspidine based on in vivo and ex vivo data from CD-1 mice after i.v. injection. The biokinetic data was obtained either by dynamic hybrid small animal PET/MR imaging or by an organ harvesting approach in mice followed by extrapolation to the human scale. Subsequently, the ODs were estimated with OLINDA and the ED calculated using tissue weighting factors published by ICRP 60 [26] and ICRP 103 [27]. Finally, we performed a first-in-human dosimetry study of (*S*)-(−)-[^18^F]fluspidine in four healthy volunteers confirming the radiation safety of that promising radioligand.

### 2.1. Human Dosimetry Estimation from Small Animal PET/MRI and Biodistribution Studies

**Figure 1:**
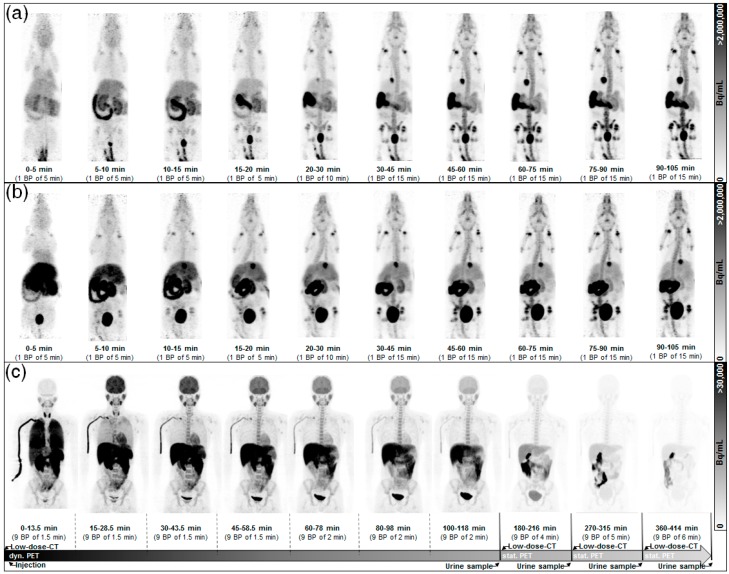
Representative time series (MIP) of mice (**a**), (**b**), and a volunteer (**c**) after i.v. injection of (*S*)-(−)-[^18^F]fluspidine (**a**), (**c**) and (*R*)-(+)-[^18^F]fluspidine (**b**). Furthermore, the diagram shows the scan protocol for humans clarifying the dynamic and static PET part as well as the urine voiding intervals.

Representative dynamic PET images in mice obtained at different times p.i. of (*S*)-(−)-[^18^F]fluspidine and (*R*)-(+)-[^18^F]fluspidine are shown in Figure 1. A high initial uptake of activity in liver, small intestines, and gallbladder wall as well as a fast clearance during the investigation time was observed. Exemplary time-activity curves (TACs) with fitting functions to calculate the numbers of disintegration (please see Section 4.4) for (*S*)-(−)-[^18^F]fluspidine and (*R*)-(+)-[^18^F]fluspidine are presented in Figure S1. The corresponding mean uptake values (in terms of % ID at a particular time p.i.; Tables S3 and S4) reflect lower values of the S-enantiomer in comparison to the R-enantiomer.

The biodistribution study confirmed the enantiomer-specific performance (Figure S2). The decrease of the %ID values of (*S*)-(−)-[^18^F]fluspidine during the course of the study (Table S1) is contrasted by a stagnation of the washout of activity after administration of (*R*)-(+)-[^18^F]fluspidine (Table S2), which is most obvious in brain, spleen, kidneys, and lung. Accordingly, animal PET and biodistribution data revealed higher ODs and EDs for (*R*)-(+)-[^18^F]fluspidine compared to (*S*)-(−)-[^18^F]fluspidine (Table 1 and Table 2). We estimated the highest OD values for (*S*)-(−)-[^18^F]fluspidine and (*R*)-(+)-[^18^F]fluspidine from animal PET/MRI in urinary bladder, kidneys, spleen, gallbladder wall, and liver (Table 1). From animal organ harvesting derived biodistribution, the highest values were estimated in kidneys, upper large intestine, small intestine, and lungs (Table 2).

For (*S*)-(−)-[^18^F]fluspidine we estimated the ED in humans from animal PET/MRI and organ harvesting derived biodistribution to be 12.9 ± 0.4 μSv/MBq and 14.0 ± 0.5 μSv/MBq, respectively, and for (*R*)-(+)-[^18^F]fluspidine to be 16.7 μSv/MBq and 18.4 μSv/MBq, respectively Accordingly, for (*R*)-(+)-[^18^F]fluspidine the ED is higher than for (*S*)-(−)-[^18^F]fluspidine in both experimental conditions; however, statistical significance could be calculated only for the imaging-derived data (*p* = 0.025, students *t* test, *n* = 3/group). For the organ harvesting study, a *t* test is not applicable due to methodical reasons.

Detailed biokinetic data expressed as mean %ID of the mice organ harvesting or imaging method can be found in the supplemental material (Tables S1–S4).

### 2.2. Human Dosimetry from the First-in-Human Study

There were no adverse effects reported in any of the four volunteers after i.v. injection of (*S*)-(−)-[^18^F]fluspidine, and no significant changes in vital signs were monitored.

Typical TACs and fitted curves are shown in Figure S3. The results of the dose assessment are presented in Table 3. Detailed biokinetic data expressed as mean %ID of the clinical study can be found in the supplemental material (Table S5).

The highest OD values for (*S*)-(−)-[^18^F]fluspidine were estimated in gallbladder wall, small intestine, stomach, and kidneys. The effective dose of (*S*)-(−)-[^18^F]fluspidine for humans was estimated to be 21.0 ± 1.3 μSv/MBq. A summary of the ED estimates for both enantiomers of [^18^F]fluspidine, the different methods and species can be found in Table 4.

The toxicity results (please see supplemental methods and results) of the pathologic examination in Wistar rats indicated that (*S*)-(−)-fluspidine after single intravenous administration did not cause toxicological changes in pathological and histopathological parameters on day 2 and day 15. The no observed effect level (NOEL) of (*S*)-(−)-fluspidine after single intravenous administration in this study for both day 2 and day 15 was determined to be 620 μg/kg (highest tested dose).

## 3. Discussion

With this study, we support the clinical translation of the novel radiotracer [^18^F]fluspidine for imaging of σ_1_ receptors by preclinical and clinical radiation dosimetry studies. We have derived internal radiation dosimetry of the enantiomers (*S*)-(−)-[^18^F]fluspidine and (*R*)-(+)-[^18^F]fluspidine by organ harvesting and dynamic small animal PET/MR imaging in mice and compared the results of both methods with each other. Finally, we performed a clinical study to calculate radiation doses for humans following intravenous injection of (*S*)-(−)-[^18^F]fluspidine and to validate the results achieved by the animal dose assessment. The main findings are (i) methodical issues regarding radiation estimates for humans extrapolated from small animals; (ii) radiation dose differences between the two enantiomers (*S*)-(−)-[^18^F]fluspidine and (*R*)-(+)-[^18^F]fluspidine; and (iii) confirmation of the radiation safety of (*S*)-(−)-[^18^F]fluspidine for clinical studies.

We would like to point out that both the preclinical as well as the clinical studies have shown that the novel σ_1_ receptor imaging agent (*S*)-(−)-[^18^F]fluspidine fulfils the requirements regarding radiation dose in human clinical trials, although in comparison to the extrapolated animal data a 1.6-fold higher value (*p* < 0.001, students *t* test, *n* = 3/group) of the actual ED has been calculated from the human study.

The main reasons for this discrepancy are assumed to be related to several methodological shortcomings of the extrapolation procedures. One deficiency is the assumption in the adult male model implemented in OLINDA 1.0, that the anatomical organ arrangement between mice and humans is identical. However, a simple mass extrapolation in animals and using a human phantom that does not take into account the spatial interactions of the organs in comparison to mouse (reflected by the *S*-values), is insufficient. A novel approach using the implementation of rodent specific dosimetry models in OLINDA 2.0 [33] remains to be assessed. Another limitation belongs to the extrapolation methods used to adapt the animal time scale and uptake scale. The currently most qualified methods [34] cancel out at least partially species differences in metabolism as well as body and organ weight. However, a compensation for species-specific differences in the tracer uptake, i.e., differences in the expression of the target in the respective organ, is not possible. Furthermore, the aspect of the effect of significant size differences between the species on dose estimations has been recently addressed by our group during the clinical translation of a radioligand for imaging of nicotinic acetylcholine receptors by directly comparing dosimetry in piglets (~15 kg) and humans [24,25]. However, an underestimation of the radiation dose in humans of about 40% remained. Hence, a simple size-dependent effect is not likely, as reflected by the findings of Zanotti-Fregonara et al. [16]. In this study, both under- and overestimations of the effective dose in humans, ranging from −11% to +72%, by using biokinetic data for nine PET tracers obtained in monkeys are reported (baboons and rhesus monkeys, weight: 9.9 ± 3.6 kg). Altogether, these findings clearly indicate the need to take species-specific pharmacokinetics into account of both the radiotracer and radiometabolites as they potentially result in significant deviations in the dosimetry of the radiotracer under investigation.

The direct comparison between the two preclinical methods of dose estimation via organ harvesting and dynamic small animal PET imaging reveals negligible differences regarding ED values of the respective [^18^F]fluspidine enantiomer under investigation. However, for both radiotracers, slightly lower organ doses were detected in the imaging than in the organ harvesting approach. This outcome is most likely related to anesthesia-mediated effects on hemodynamics and metabolism [35,36], although based on the currently available data no mechanistic explanation can be provided. The attractive approach reported by Bretin et al. [14] to compensate for deviations between these two preclinical methods by correcting the image derived TACs according to the activity values measured ex vivo by gamma-counting after scanning is not applicable here, because in contrast to our study they used anesthetized animals for the organ harvesting method as well.

Another interesting finding in our preclinical study is that although both enantiomers accumulate specifically in σ_1_ receptor rich regions in the brain [9], they exhibit pronounced differences in their ED values. This is most probably related to marked differences in their pharmacokinetics and pharmacology [9]. The TACs of (*S*)-(−)-[^18^F]fluspidine and (*R*)-(+)-[^18^F]fluspidine in mice, obtained by either organ harvesting or PET imaging, are different in terms of maximal uptake value (in %ID) and the shape of the curve. Hence, slower elimination rates, up to 1.3-fold higher OD values and subsequently higher ED values (*p* = 0.025, students *t* test; PET imaging approach with *n* = 3/group) were observed for the (*R*)-(+)-enantiomer. Following an initial washout, detected for both enantiomers, the elimination of activity stagnates in nearly all organs after administration of (*R*)-(+)-[^18^F]fluspidine. This corresponds to the enantioselective tracer kinetics already observed in most regions of the pig brain and the significantly higher BP_nd_ values of (*R*)-(+)-[^18^F]fluspidine [9]. Assuming such enantioselective pharmacokinetics for other tissues as well due to the expression of σ_1_ receptors in almost all tissues [3,37], the slower washout of (*R*)-(+)-[^18^F]fluspidine from the organ tissues was to be expected. Statistical significance in terms of ED was attained solely with the imaging-derived data because only with this approach a complete set of biokinetic data of one animal and hence individual OD and ED values are available. By contrast, no individual time-activity data can be obtained from ex vivo biodistribution studies because each animal contributes to only a single OD value.

The strong correlation between pharmacokinetics and ED values is demonstrated also by a comparison of the herein investigated enantiomers of [^18^F]fluspidine with the enantiomers of [^18^F]flubatine, a ligand for α4β2 nicotinic acetylcholine receptors [24,25]. Our preclinical and clinical dosimetry studies of (+)-[^18^F]flubatine and (−)-[^18^F]flubatine, both possessing very similar biokinetics in different species up to humans, revealed no significant differences in the ED between the two enantiomers. No significant differences were observed also regarding the ED values of the enantiomers of [^11^C]mirtazapine, although the enantioselectivity of the OD values estimated for brain corresponds with the enantioselectivity of the brain kinetics [38]. Altogether, findings on either different or comparable ED values of enantiomers of chiral compounds used as PET imaging agents strongly reflect the influence of enantioselective processes during their interaction with the chiral compounds in biological systems such as receptor proteins or metabolizing enzymes [39].

Although the ED values of both enantiomers of [^18^F]fluspidine show a 1.6 fold difference, the excretion route of ^18^F is similar. A renal/hepatobiliary clearance can be assumed from the two preclinical models due to a high uptake of activity in the intestinal and hepatobiliary as well as renal tract, which results in comparatively high OD values in the liver, gallbladder wall, small intestine, kidneys, and urinary bladder. Furthermore, in fully awake animals used in the organ harvesting distribution study the urinary bladder is less exposed to radiation than in anesthetized mice due to urinary retention under isoflurane narcosis [40,41,42,43].

Based on the preclinical biokinetic data shown herein as well as in our recent PET study using piglets [9], different clinical applications came into consideration for the two enantiomers of [^18^F]fluspidine. The relatively slow kinetics and nearly constant activity accumulation of (*R*)-(+)-[^18^F]fluspidine in the observed organs and tissues which might translate into high signal-to-noise ratios in σ_1_ expressing tumors and metastases makes this enantiomer interesting for cancer imaging. By contrast, the (*S*)-(−)-enantiomer provided favorable properties for neuroimaging and data analysis with a special regard to kinetic modeling due to the high initial brain uptake and fast washout and was therefore selected for a first-in-human study. The radiation dose of (*S*)-(−)-[^18^F]fluspidine in human tissues has been estimated after injection of the radiotracer in two female and two male healthy volunteers. The hereby obtained TACs (presented in Figure S3) confirmed the assumed renal/hepatobiliary clearance. The radioligand was rapidly removed from brain, stomach, liver, and spleen within one hour post injection, while a slower clearance from red marrow, already observed in earlier σ_1_ receptor ligand studies [44,45], reflects the high expression of σ_1_ receptors in rapidly dividing tissues. Hence, it was proposed that σ_1_ receptor ligands may also be used as proliferation markers [46]. The effective dose of the σ_1_ receptor ligand (*S*)-(−)-[^18^F]fluspidine is 21.0 μSv/MBq, well within the range of other ^18^F-labeled diagnostic radiotracers (Table 4). Thus, in combination with a NOEL of at least ~600 μg/kg, the application of (*S*)-(−)-[^18^F]fluspidine as PET imaging agent in humans is safe.

## 4. Materials and Methods

The time-dependent radioactive data for the animal and human studies was acquired with three different techniques. (i) The mice were scanned in a preclinical small animal PET/MRI while the (ii) human study was performed on a clinical PET/CT system. In addition the (iii) ex vivo biodistribution study in mice was performed by post mortem organ dissection followed by counting for radioactivity in a gamma counter.

### 4.1. Synthesis of [^18^F]Fluspidine

The synthesis of (*S*)-(−)-[^18^F]fluspidine for the human application was performed as described by Fischer et al. [8] with minor modifications. Briefly, the tracer was produced by phase transfer catalyst assisted nucleophilic substitution (100 °C, 15 min) using a precursor molecule with a tosyl-leaving group (2 mg in 1 mL dry CH_3_CN). Purification and formulation was achieved by semipreparative HPLC and solid phase extraction, respectively. Overall synthesis time was 50 min, radiochemical purity exceeded 97% and specific activity was determined to be 230 ± 160 GBq/μmol (*n* = 16 syntheses).

For the animal studies, enantiomerically pure (*S*)-(−)-[^18^F]fluspidine and (*R*)-(+)-[^18^F]fluspidine was prepared on a TRACERlab FX F-N synthesizer (GE Healthcare) as described in previous publications [9,47]. The radiochemical purity of (*R*)-(+) or (*S*)-(−)-[^18^F]fluspidine was >99%, and the specific activity at the end of the synthesis was 650 and 870 GBq/μmol, respectively [48].

### 4.2. Preclinical Dosimetry Studies

All animal experiments were approved by the responsible institutional and federal state authorities (Landesdirektion Leipzig; TVV 08/13). A toxicological study was confirmed and can be found in the supplemental material.

#### 4.2.1. Ex Vivo Biodistribution Study (Organ Harvesting Method)

Female CD-1 mice (age: 12 weeks) received an intravenous injection of (*R*)-(+)-[^18^F]fluspidine (0.35 ± 0.08 MBq; weight: 29.8 ± 2.2 g; *n* = 28) or (*S*)-(−)-[^18^F]fluspidine (0.39 ± 0.05 MBq; weight: 29.3 ± 1.9 g; *n* = 22). Two to three animals per time point were sacrificed by cervical dislocation at 5, 15, 30, 45, 60, 90, 120, 180, and 240 min. p.i. The brain, heart, lung, stomach, small intestine (SI), large intestine (LI), liver, kidneys, urinary bladder (UB), spleen, thymus, pancreas, adrenals, and ovaries were dissected, weighed, and the accumulated activity quantified in a gamma-counter (WIZARD Automatic Gamma Counter, PerkinElmer, Waltham, MA, USA) to determine the percentage injected activity (dose) per gram of tissue (%ID/g).

In addition, the sampling time p.i. and %ID/g values were scaled proportionately to human magnitude (please see Equations (1) and (2) in Section 4.4) prior to dose estimation with OLINDA/EXM (Vanderbilt University, Nashville, TN, USA, version 1.0).

#### 4.2.2. In Vivo Imaging Based Study (Imaging Method)

The animals were initially anesthetized with 4% of isoflurane and were positioned prone in a small-animal PET/MRI system (nanoScan^®^ PET/MRI, MEDISO, Budapest, Hungary) equipped with respiratory monitoring, heated mouse bed (37 °C), and inhalation anesthesia (1.8% isoflurane in a 60% oxygen/40% air gas mixture at 250 mL/min airflow; Anaesthesia Unit U-410, agntho’s, Lidingö, Sweden; Gas blender 100 series, MCQ Instruments, Rome, Italy). Prior to the PET scan, a scout image MR sequence was done to outline the animal dimensions. Female CD-1 mice (age: 12 weeks, weight: 30.9 ± 1.3 g) were injected via the tail vein with (*S*)-(−)-[^18^F]fluspidine (13.2 ± 3.0 MBq; *n* = 3) or (*R*)-(+)-[^18^F]fluspidine (12.6 ± 1.4 MBq; *n* = 3) in a volume of 200 μL saline. The injected dose was calculated by the difference of the radioactivity in the syringe before and after the injection. A dynamic whole body animal PET scan of 105 min length (Figure 1) was started simultaneously. This scanning time was chosen to represent the protocol of the human study (after time extrapolation according to Equation (2)) based on a priori biokinetic information from the ex vivo investigation. Following the PET scan, a 20 min T1-weighted whole body MR scan (gradient echo sequence, T_R_ = 20 ms; T_E_ = 3.2 ms) was performed for anatomical orientation after co-registration and attenuation correction at the reconstruction step.

### 4.3. First-in-Human Dosimetry Study (Imaging Method)

The first-in-human use of (*S*)-(−)-[^18^F]fluspidine was authorized by the competent authorities in Germany, the Federal Institute for Drugs and Medical Devices (Bundesamt für Arzneimittel und Medizinprodukte, BfArM) and the Federal Office for Radiation Protection (Bundesamt für Strahlenschutz, BfS) as well as by the local ethics committee and was conducted in accordance with the Declaration of Helsinki. Informed consent was obtained from four healthy volunteers (2 f, 2 m; age: 23 ± 3 years; weight: 56 ± 4 kg). The volunteers were positioned supine with the arms down in a clinical PET/CT system (Biograph 16, Siemens, Erlangen, Germany) and received an intravenous injection of 255 ± 9 MBq (*S*)-(−)-[^18^F]fluspidine. Simultaneously, the PET scan was started. It was divided into a dynamic part up to 2 h p.i. (7 frames) and a static part up to 7 h p.i. (3 frames) with increasing time per bed position (from 1.5 up to 6 min) as shown in Figure 1. The volunteers left the investigation table four times to stretch out. All urine was collected, weighed, and the activity was determined in a gamma-counter (Packard Cobra II 5003 Auto Gamma Counting System, GMI, Ramsey, MN, USA) cross calibrated to the PET/CT system.

### 4.4. Image Reconstruction and Analysis of the Preclinical and Clinical Data

The PET images were iteratively (ordered-subsets expectation maximization, OSEM) reconstructed (preclinical: 4 iterations, 6 subsets; clinical: 4 iterations, 8 subsets) and corrected for decay, randoms, scatter, and dead time, μ-maps for attenuation correction of PET-emission data were derived from the CT or MR [49] structural data, respectively. The PET data were re-binned into 10 time frames (preclinical: 4 × 5 min, 1 × 10 min, and 5 × 15 min; clinical: 4 × 12 min, 3 × 16 min, 1 × 32 min, 1 × 40 min, and 1 × 48 min), and the reconstructed PET/MRI and PET/CT images were co-registered manually with ROVER (ABX, advanced chemical compounds, Radeberg, Germany, version 2.1.17). Quantitative evaluation was performed by drawing volumes of interest (VOI) for brain, gallbladder, large intestine, small intestine, stomach, heart, kidneys, liver, lungs, pancreas, red marrow (backbone, pelvis, sternum), spleen, thyroid, testes, and urinary bladder (Figure S4). The PET derived biokinetic data is expressed as percentage of injected activity (dose) per cubic centimeter (%ID/cm^3^).

For human dosimetry estimation from animal biodistribution and PET/MR imaging, animal organ masses and time scale was extrapolated to human magnitudes [20,30]. At first, the organ-specific animal %ID data were extrapolated to the human scale with the equation

(1) $$\frac{\%ID}{{organ}_{human}}=\frac{\%ID}{g_{mouse}}\;\cdot \;m_{{organ}_{human}}\;\cdot \;\frac{m_{mouse}}{m_{human}}$$

with the fraction of the injected activity in the corresponding human organ = $\frac{\%ID}{organ_{human}}$, the fraction of injected activity per gram animal organ tissue = $\frac{\%ID}{g_{mouse}}$ and $m_{organ_{human}}$ the mass of the corresponding human organ [50]. At second, a time scale extrapolation is needed due to differences in the metabolic rate using the equation

(2) $$t_{human}=t_{mouse}{\left\{\frac{m_{human}}{m_{mouse}}\right\}}^{0.25}$$

including the human time scale = $t_{human}$, the animal time scale = $t_{mouse}$ and the ratio of animal and human body weights = $\frac{m_{mouse}}{m_{human}}$. The allometric coefficient of 0.25 generally describes the differences between the two species regarding physiological processes such as biological half-life [20,50,51]. Hence, using this time extrapolation approach with an exponent of 1/4, a 105 min PET scan in mice is sufficient to represent 10 h in humans (Figure S1).

The human dosimetry estimation was performed with the data extrapolated as well as the genuine human data using OLINDA/EXM software [33]. The time-activity curves were estimated by exponential fitting and calculating the time integral, which equals the number of disintegrations (NODs) per organ during the observation period normalized to 1 Becquerel administered activity dose. Due to narcosis, mice did not void urine during the imaging session. Therefore, activity data of the urinary bladder was derived from the image for each time point. In contrast, for humans the activity concentration data of the urinary bladder was obtained in a more direct approach. At first, the activity and volume of urine was determined in the last frame of the PET scan before each micturition. Afterwards, the voided urine was collected, weighed, and the activity of three aliquots (assuming 1 mL = 1 g) determined with a gamma counter, and the activity of the whole sample estimated. The difference between imaged and sampled urine activity is equal to the residue of radioactive urine in the urinary bladder. To calculate the NOD in the human urinary bladder, the time-activity curve is integrated using a trapezoidal equation

(3) $$\%{\tilde{ID}}_{UB}=\frac{1}{2}\overset{n-1}{\underset{i=1}{\sum }}\left(\%ID_{i}+\%ID_{i+1}\right)\left(t_{i+1}-t_{i}\right)$$

with the fraction of injected activity *%ID_i_* at the time *t_i_* and the cumulated activity of the urinary bladder i.e., the NOD $\%{\tilde{ID}}_{UB}$.

Furthermore, the NODs of the gastric system were calculated following the ICRP GI model (ICRP 30) as implemented in OLINDA 1.0. The NODs obtained either from the EXM module or the trapezoidal equation were transferred to OLINDA. The OD for the chosen hermaphroditic adult male phantom is estimated following the MIRD scheme [52]. The *S* values [53,54] are pre-calculated and implemented for the respective phantom in OLINDA. Subsequently, the ED contribution from each organ is calculated by multiplying the ODs with the respective tissue weighting factors as published by the International Commission on Radiological Protection (ICRP 103 [27]) for each organ. As these weighting factors require the ICRP 110 phantom [55] which is not available in OLINDA version 1.0, the ED results by using the tissue weighting factors published by ICRP 60 [26] were estimated in addition (Table 1, Table 2 and Table 3).

## 5. Conclusions

The results achieved from this study support the potential of (*S*)-(−)-[^18^F]fluspidine as a clinically applicable PET imaging agent for the investigation of σ_1_ receptors. As shown before, the extrapolation of preclinical data obtained by dosimetry studies in small animals by either organ harvesting or PET imaging results in an underestimation of the human ED values most due to limitations in allometric scaling and species-specific target expression. However, the imaging approach excels in comparison to the organ harvesting method for obtaining extensive whole body kinetic information using a significantly reduced number of animals. Thus, small animal image based dosimetry is recommended as the preferable method for preclinical dose estimates prior to the application for first-in-human studies. However, preclinical dose estimates remain preliminary and need to be confirmed in human studies.

While we are presently evaluating the utility of (*S*)-(−)-[^18^F]fluspidine for quantification of pathological changes in the expression of σ_1_ receptors in major depressive disorder, the entire potential of the enantioselective pharmacokinetics of (*S*)-(−)-[^18^F]fluspidine and (*R*)-(+)-[^18^F]fluspidine for imaging of σ_1_ receptors in neuropsychiatric, neuro-oncological, and oncological diseases remains to be further investigated.

## Figures and Tables

**Table 1:** OD and ED in μSv/MBq based on the imaging method with a small animal PET/MRI. ODs calculated for the adult male model (73.7 kg, implemented in OLINDA) based on mouse biodistribution and organ geometry data that were scaled proportionately to human circumstances.

Target Organ	(*S*)-(−)-[^18^F]Fluspidine				(*R*)-(+)-[^18^F]Fluspidine			
	OD	SD	ED Contr.	SD	OD	SD	ED Contr.	SD
Adrenals	10.50	0.74	0.09	0.01	11.00	1.55	0.09	0.01
Brain	10.10	2.34	0.10	0.02	13.20	1.19	0.13	0.01
Breasts	5.93	0.10	0.71	0.01	6.19	1.77	0.74	0.21
Gallbladder Wall	25.60	9.57	0.22	0.08	30.10	11.90	0.26	0.10
LLI Wall	14.00	1.48	0.84	0.09	13.80	1.39	0.83	0.08
Small Intestine	23.10	3.22	0.20	0.03	22.60	1.92	0.20	0.02
Stomach Wall	10.50	0.60	1.26	0.07	12.70	1.10	1.52	0.13
ULI Wall	20.50	4.96	1.23	0.30	25.60	2.19	1.54	0.13
Heart Wall	9.85	0.60	0.08	0.01	10.50	1.31	0.09	0.01
Kidneys	37.60	14.80	0.32	0.13	26.90	2.74	0.23	0.02
Liver	25.00	3.23	1.00	0.13	26.10	4.65	1.04	0.19
Lungs	10.40	2.30	1.25	0.28	10.80	0.89	1.30	0.11
Muscle	7.57	0.07	0.07	0.00	7.86	1.96	0.07	0.02
Ovaries	11.50	0.82	0.92	0.07	11.90	1.95	0.95	0.16
Pancreas	10.90	0.69	0.09	0.01	24.80	1.79	0.21	0.02
Red Marrow	10.80	0.37	1.30	0.04	12.80	1.27	1.53	0.15
Osteogenic Cells	12.70	0.13	0.13	0.00	14.00	3.18	0.14	0.03
Skin	5.61	0.02	0.06	0.00	5.82	1.73	0.06	0.02
Spleen	26.10	7.29	0.22	0.06	31.80	20.00	0.27	0.17
Testes	7.46	0.39	0.00	0.00	7.63	2.04	0.00	0.00
Thymus	7.19	0.11	0.06	0.00	7.52	2.21	0.06	0.02
Thyroid	7.61	1.09	0.30	0.04	10.10	0.35	0.41	0.01
Urinary Bladder Wall	58.00	15.90	2.32	0.64	55.70	19.30	2.23	0.77
Uterus	12.80	1.28	0.11	0.01	13.00	1.49	0.11	0.01
Total Body	8.68	0.14	0.00	0.00	9.13	1.67	0.00	0.00
ED			12.9	0.4			14.0	0.5
ED ICRP 60			14.8	1.7			15.2	1.9

OD = organ dose; ED contr. = effective dose contribution; SD = standard deviation, mean over 3 animals; LLI = large lower intestine; ULI = upper large intestine.

**Table 2:** OD and ED in μSv/MBq based on mouse organ harvesting after dissection and organ counting in a gamma-counter. The organ geometry data were scaled proportionately to human circumstances. ODs calculated for the adult male model (73.7 kg, implemented in OLINDA).

Target Organ	(*S*)-(−)-[^18^F]Fluspidine		(*R*)-(+)-[^18^F]Fluspidine	
	OD	ED Contr.	OD	ED Contr.
Adrenals	36.0	0.3	18.6	0.2
Brain	12.4	0.1	12.6	0.1
Breasts	11.2	1.3	11.3	1.4
Gallbladder Wall	15.5	0.1	14.0	0.1
LLI Wall	19.0	1.1	16.4	1.0
Small Intestine	31.9	0.3	25.1	0.2
Stomach Wall	14.8	1.8	14.3	1.7
ULI Wall	33.3	2.0	25.6	1.5
Heart Wall	17.9	0.2	22.3	0.2
Kidneys	35.6	0.3	27.6	0.2
Liver	12.5	0.5	10.3	0.4
Lungs	30.5	3.7	45.5	5.5
Muscle	7.2	0.1	7.1	0.1
Ovaries	17.0	1.4	24.9	2.0
Pancreas	26.2	0.2	21.7	0.2
Red Marrow	13.6	1.6	13.5	1.6
Osteogenic Cells	19.6	0.2	19.1	0.2
Skin	9.1	0.1	8.7	0.1
Spleen	17.6	0.2	17.2	0.1
Testes	11.2	-	10.8	-
Thymus	12.0	0.1	19.3	0.2
Thyroid	11.7	0.5	11.5	0.5
Urinary Bladder Wall	13.9	0.6	20.2	0.8
Uterus	15.9	0.1	14.9	0.1
Total Body	12.5	-	12.2	-
ED		16.7		18.4
ED ICRP 60		17.3		20.1

OD = organ dose; ED contr. = effective dose contribution; LLI = large lower intestine; ULI = upper large intestine.

**Table 3:** First-in-human data, OD and ED in μSv/MBq. The ODs were calculated for the adult male model (73.7 kg, implemented in OLINDA) based on human biodistribution and organ geometry data.

Target Organ	(*S*)-(−)-[^18^F]Fluspidine			
	OD	SD	ED Contr.	SD
Adrenals	15.3	1.1	0.1	0.0
Brain	22.6	4.2	0.2	0.1
Breasts	6.5	0.5	0.8	0.1
Gallbladder Wall	60.7	10.6	0.5	0.1
LLI Wall	16.6	5.1	1.0	0.3
Small Intestine	56.9	10.6	0.5	0.1
Stomach Wall	31.5	3.3	3.8	0.4
ULI Wall	24.3	5.2	1.5	0.3
Heart Wall	17.7	1.3	0.2	0.0
Kidneys	31.1	5.2	0.3	0.0
Liver	76.0	17.7	3.0	0.4
Lungs	28.2	2.9	3.4	0.3
Muscle	7.8	0.5	0.1	0.0
Ovaries	13.8	1.0	1.0	0.5
Pancreas	15.9	0.7	0.1	0.0
Red Marrow	23.2	2.2	2.8	0.1
Osteogenic Cells	18.0	1.6	0.2	0.0
Skin	5.3	0.5	0.1	0.0
Spleen	24.0	4.2	0.2	0.0
Testes	8.0	2.6	0.8	0.4
Thymus	7.5	0.7	0.1	0.0
Thyroid	8.4	1.4	0.3	0.1
Urinary Bladder Wall	24.7	3.4	1.0	0.1
Uterus	13.0	0.7	0.1	0.1
Total Body	11.4	0.3	0.0	0.0
ED			21.0	1.3
ED ICRP 60			22.1	0.8

OD = organ dose; ED contr. = effective dose contribution; SD = standard deviation, mean over four volunteers; LLI = large lower intestine; ULI = upper large intestine.

**Table 4:** Comparison of dosimetry results (ED) for different PET tracers including the current study with [^18^F]fluspidine.

Tracer	Target Organ	Clinical (μSv/MBq)	Preclinical (μSv/MBq)	Reference
(*S*)-(−)-[^18^F]fluspidine	brain, tumor	21.0	12.9 (mouse, imaging) 16.7 (mouse, harvesting)	this study
(*R*)-(+)-[^18^F]fluspidine	brain, tumor	n.a.	14.0 (mouse, imaging) 18.4 (mouse, harvesting)	this study
(−)-[^18^F]flubatine (formerly [^18^F]NCFHEB)	brain	23.4	12.5 (mouse) 14.7 (piglet, imaging)	[24]
(+)-[^18^F]flubatine	brain	23.0	12.1 (mouse, imaging) 14.3 (piglets, imaging)	[25]
[^18^F]FEDAA1106	brain	36	21.0 (male mouse) 26.0 (female mouse)	[28]
[^18^F]FET	brain tumor	16.5	9.0	[29,30]
2-[^18^F]F-A85380	brain	19.4	n.a.	[31]
[^18^F]FDG	multiple	19.0	n.a.	[32]

## Supplementary Materials

Supplementary materials can be accessed at: http://www.mdpi.com/1420-3049/21/9/1164/s1.
