# Supplementary material for: Evaluation of the Enantiomer Specific Biokinetics and Radiation Doses of [18F]Fluspidine—A New Tracer in Clinical Translation for Imaging of σ1 Receptors
Source: Molecules. 2016 Sep 1;21(9):1164. doi: 10.3390/molecules21091164 (PMC6273209; doi:10.3390/molecules21091164)
Supplement: Supplementary file 1 [file molecules-21-01164-s001.pdf]

Supplementary Materials: Evaluation of the Enantiomer Specific Biokinetics and Radiation Doses of [^{18}F]Fluspidine—A New Tracer in Clinical Translation for Imaging of σ$_1$ Receptors

Mathias Kranz, Bernhard Sattler Nathanael Wüst, Winnie Deuther-Conrad, Marianne Patt, Philipp M. Meyer, Steffen Fischer, Cornelius K. Donat, Bernhard Wünsch, Swen Hesse, Jörg Steinbach, Peter Brust and Osama Sabri

Supplemental Methods and Results

Animal Toxicity Study of (S)-(−)-Fluspidine

The purpose of this study was to assess the acute toxicity of (*S*)-(−)-fluspidine when administered by a single intravenous injection to rats followed by an observation period of 2 or 15 day (in accordance with EU cGLP). Briefly, the study was performed with 4 test groups, including 1 control (saline and dimethylsulfoxide) and 3 dose groups (6.2, 62 and 620 µg/kg, (*S*)-(−)fluspidine), with Wistar rats (60 m, 60 f, 12 weeks) divided into two experiments: day 2 (40 males and 40 females) and day 15 (20 males and 20 females). The animals were weighted and allocated to the test groups based on their actual body weights. The animals were sacrificed at two time periods (day 2 and day 15 after test item administration) and then examined macroscopically and histopathologically. In addition the following parameters were evaluated: mortality, clinical observation (spasms, tremor and hyperactivity), body weights, food consumption, haematology (white blood cells, red blood cells, hemoglobin, hematocrit, mean corpuscular volume, mean corpuscular hemoglobin, platelets, lympohcytes, neutrophils, eosinophils, basophils, monocytes), clinical chemistry (alkaline phosphatase, aspartate aminotransferase, alanine aminotransferase, glucose, cholesterol, triacylglycerols, creatinine, urea, bilirubin, albumin, calcium, phosphorus, sodium, potassium chloride), pathology and histopathology.

No statistically significant differences in body weights between control and treated groups in either gender were detected. The food consumption corresponded with body weight development. All haematology parameter′s values on day 2 and day 15 were within physiological range for this species. Individual divergences of some values of the haematology parameters were slight and not connected with treatment. No test item effect on the haematology parameters were observed in this study. There were no findings in clinical chemistry parameters which could be definitively considered as adverse. The average values of all test groups were within the historical control ranges. Occasional changes had no dose relationship, they were considered to be a result of intraindividual and interindividual variability for this species. The results of pathology examination indicated that (−)-Fluspidine after single intravenous administration did not cause toxicological changes in pathological and histopathological parameters on day 2 and day 15. The no observed effect level (NOEL) of (*S*)-(−)-fluspidine after single intravenous administration in this study for both day 2 and day 15 was determined to be 620 µg/kg.

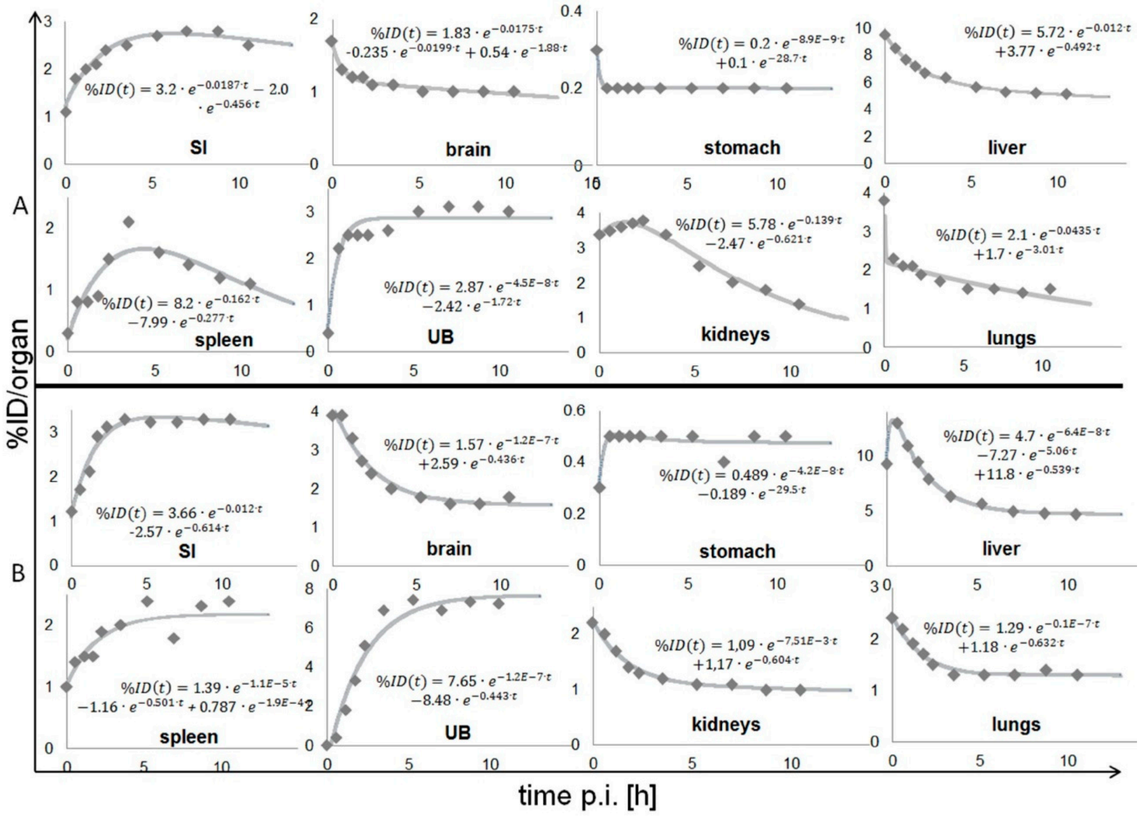

Figure S1. Exemplary time-activity curves with fitting functions of one female CD-1 mouse using the imaging method for, (**A**) (*S*)-(−)-[^{18}F]fluspidine and (**B**) (*R*)-(+)-[^{18}F]fluspidine. Abbreviations: SI, small intestine; UB, urinary bladder.

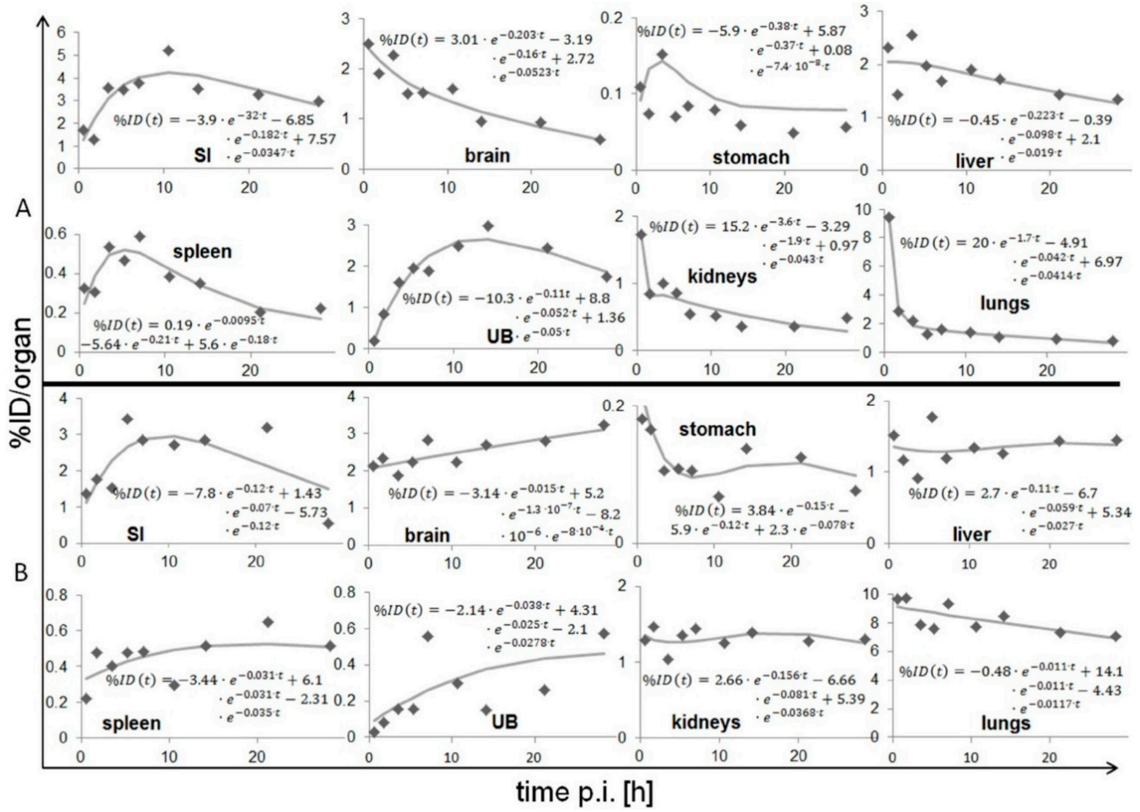

Figure S2. Mean time-activity curves for 22/28 mice using the organ harvesting method after i.v. injection of (**A**) (*S*)-(−)-[^{18}F]fluspidine and (**B**) (*R*)-(+)-[^{18}F]fluspidine. Abbreviations: SI, small intestine; UB, urinary bladder.

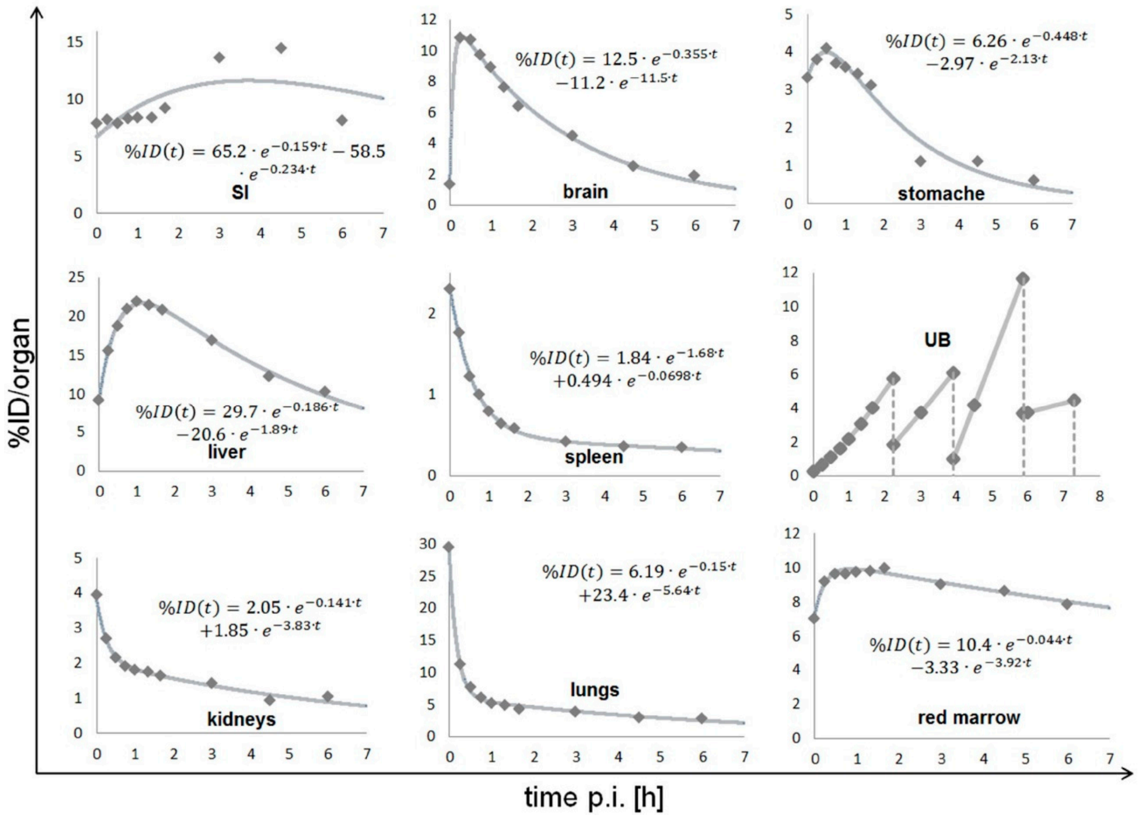

Figure S3. Exemplary time-activity curves with fitting functions of one volunteer using the imaging method and a clinical PET/CT after injection of (*S*)-(−)-[^{18}F]fluspidine. Abbreviations: SI, small intestine; UB, urinary bladder.

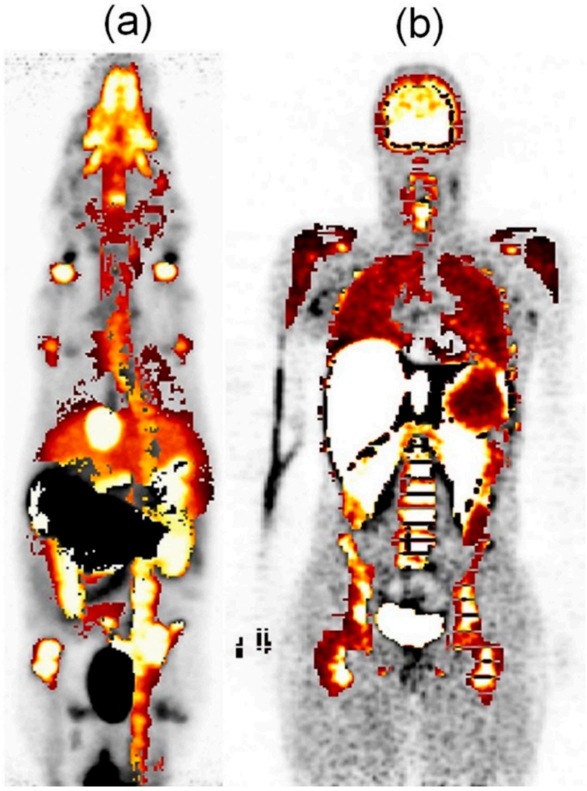

Figure S4. Summed PET images (MIP) with VOIs highlighted for a (**a**) female CD-1 mouse (2 h) and (**b**) female volunteer (90 min) after i.v. injection of (*S*)-(−)-[^{18}F]fluspidine.

Table S1. Mean %ID values of 22 female CD-1 mice after i.v. injection of 0.39 ± 0.05 MBq (*S*)-(−)-[^{18}F]fluspidine followed by dissection and organ counting in a gamma counter.

Organ:		Adrenals			Brain			LLI			SI			Stomach			ULI		
Modell		Animal		Human	Animal		Human	Animal		Human	Animal		Human	Animal		Human	Animal		Human
Organmass (g)		0.01214		16.3	0.4736		1420	0.5604		167	2.0113		1100	1.3140		158	1.1209		220
Fractions $Time_{animal}$ (h)	$Time_{human}$ (h)	%ID/Organ	%ID/g	%ID/Organ	%ID/Organ	%ID/g	%ID/Organ	%ID/Organ	%ID/g	%ID/Organ	%ID/Organ	%ID/g	%ID/Organ	%ID/Organ	%ID/g	%ID/Organ	%ID/Organ	%ID/g	%ID/Organ
0.08	0.59	0.1	11.390	0.08	2.1	4.344	2.5	0.4	0.647	0.04	7.7	3.849	1.7	2.2	1.712	0.1	0.7	0.647	0.1
0.25	1.76	0.1	4.892	0.03	1.6	3.320	1.9	0.3	0.541	0.04	5.7	2.837	1.3	1.5	1.147	0.1	0.6	0.541	0.05
0.50	3.52	0.1	5.788	0.04	1.9	3.956	2.3	0.4	0.668	0.05	16.1	7.993	3.6	3.1	2.380	0.2	0.7	0.668	0.1
0.75	5.29	0.1	6.504	0.04	1.2	2.611	1.5	0.4	0.699	0.05	15.7	7.786	3.5	1.4	1.091	0.1	0.8	0.699	0.1
1.00	7.05	0.1	8.306	0.05	1.2	2.638	1.5	0.4	0.742	0.05	17.0	8.437	3.8	1.7	1.304	0.1	0.8	0.742	0.1
1.50	10.57	0.1	4.999	0.03	1.3	2.795	1.6	0.5	0.915	0.06	23.5	11.687	5.2	1.6	1.230	0.1	1.0	0.915	0.1
2.00	14.10	0.1	4.935	0.03	0.8	1.651	0.9	0.5	0.981	0.07	15.8	7.872	3.5	1.2	0.926	0.1	1.1	0.981	0.1
3.00	21.15	0.0	3.307	0.02	0.8	1.617	0.9	0.4	0.774	0.05	14.7	7.320	3.3	1.0	0.760	0.05	0.9	0.774	0.1
4.00	28.20	0.0	3.631	0.02	0.5	1.019	0.6	0.5	0.843	0.06	13.5	6.705	3.0	1.1	0.870	0.1	0.9	0.843	0.1

Table S1. *Cont.*

Myocard			Kidneys			Liver			Lungs			Muscle			Ovaries			Pancreas		
Animal		Human	Animal		Human	Animal		Human	Animal		Human	Animal		Human	Animal		Human	Animal		Human
0.1685		316	0.3836		299	1.7656		1910	0.3115		1000	11.4298		28000	0.2885		8.71	0.1353		94.3
%ID/Organ	%ID/g	%ID/Organ	%ID/Organ	%ID/g	%ID/Organ	%ID/Organ	%ID/g	%ID/Organ	%ID/Organ	%ID/g	%ID/Organ	%ID/Organ	%ID/g	%ID/Organ	%ID/Organ	%ID/g	%ID/Organ	%ID/Organ	%ID/g	%ID/Organ
1.4	8.199	1.0	5.5	14.355	1.7	5.3	2.994	2.3	7.3	23.296	9.4	0.3	0.028	0.3	0.6	2.065	0.01	1.0	7.089	0.3
0.6	3.785	0.5	2.7	6.999	0.8	3.3	1.845	1.4	2.2	7.041	2.9	0.2	0.014	0.2	0.5	1.584	0.01	0.9	6.762	0.3
0.5	2.810	0.4	3.2	8.334	1.0	5.8	3.312	2.6	1.7	5.430	2.2	0.2	0.014	0.2	1.0	3.471	0.01	1.5	10.895	0.4
0.2	1.448	0.2	2.7	7.120	0.9	4.5	2.559	2.0	1.0	3.175	1.3	0.3	0.029	0.3	1.0	3.445	0.01	1.7	12.469	0.5
0.3	1.575	0.2	1.7	4.459	0.5	3.8	2.176	1.7	1.2	3.963	1.6	0.3	0.022	0.2	1.4	4.811	0.02	1.9	13.729	0.5
0.3	1.485	0.2	1.6	4.217	0.5	4.3	2.460	1.9	1.0	3.366	1.4	0.2	0.015	0.2	1.1	3.699	0.01	1.7	12.724	0.5
0.2	0.981	0.1	1.1	2.947	0.4	3.9	2.226	1.7	0.8	2.535	1.0	0.1	0.010	0.1	1.4	4.926	0.02	1.3	9.482	0.4
0.1	0.884	0.1	1.1	2.944	0.4	3.3	1.842	1.4	0.7	2.213	0.9	0.1	0.011	0.1	1.5	5.121	0.02	1.7	12.795	0.5
0.1	0.787	0.1	1.5	4.016	0.5	3.0	1.720	1.3	0.6	1.941	0.8	0.1	0.008	0.1	1.2	4.108	0.01	0.7	5.464	0.2

Table S1. *Cont.*

Red Marrow			Skin			Spleen			Thymus			ur. Bladder			Rem. of Body		
Animal		Human	Animal		Human	Animal		Human	Animal		Human	Animal		Human	Animal		Human
0.0392		1120	5.8869		3010	0.1069		183	0.0492		20.9	0.0529		47.6	3.7322		
%ID/Organ	%ID/g	%ID/Organ	%ID/Organ	%ID/g	%ID/Organ	%ID/Organ	%ID/g	%ID/Organ	%ID/Organ	%ID/g	%ID/Organ	%ID/Organ	%ID/g	%ID/Organ	%ID/Organ	%ID/g	%ID/Organ
0.1	1.734	0.8	0.0	0.005	0.0	0.5	4.407	0.3	0.2	3.081	0.03	0.5	9.466	0.2	64.2	17.199	79.0
0.0	1.079	0.5	0.0	0.008	0.0	0.4	4.162	0.3	0.1	1.433	0.01	2.3	44.070	0.8	77.0	20.633	88.9
0.1	2.769	1.3	0.0	0.006	0.0	0.8	7.235	0.5	0.2	3.572	0.03	4.4	83.227	1.6	58.4	15.644	83.7
0.1	3.427	1.6	0.0	0.007	0.0	0.7	6.331	0.5	0.1	2.883	0.02	5.4	101.215	2.0	62.6	16.765	85.7
0.2	4.871	2.2	0.1	0.013	0.0	0.9	7.964	0.6	0.3	6.092	0.05	5.1	97.179	1.9	61.6	16.507	84.9
0.2	4.871	2.2	0.0	0.008	0.0	0.6	5.160	0.4	0.2	3.441	0.03	6.8	129.003	2.5	53.9	14.455	83.2
0.1	3.257	1.5	0.1	0.012	0.0	0.5	4.706	0.3	0.2	4.494	0.04	8.1	153.524	3.0	62.6	16.769	86.7
0.2	5.320	2.4	0.1	0.011	0.0	0.3	2.720	0.2	0.3	5.086	0.04	6.7	125.976	2.4	66.1	17.721	87.1
0.2	5.001	2.3	0.1	0.011	0.0	0.3	2.993	0.2	0.1	2.253	0.02	4.8	90.271	1.7	70.6	18.924	88.9

Table S2. Mean %ID values of 28 female CD-1 mice after i.v. injection of 0.35 ± 0.08 MBq (*R*)-(+)-[^{18}F]fluspidine followed by dissection and organ counting in a gamma counter.

Organ:		Adrenals			Brain			LLI			SI			Stomach			ULI		
Modell		Animal		Human	Animal		Human	Animal		Human	Animal		Human	Animal		Human	Animal		Human
Organmass (g)		0.014068182		16.3	0.4776		1420	0.0879		167	1.7198		1100	0.6326		158	0.1758		220
Fractions Time$_{animal}$ (h)	Time$_{human}$ (h)	%ID/Organ	%ID/g	%ID/Organ	%ID/Organ	%ID/g	%ID/Organ	%ID/Organ	%ID/g	%ID/Organ	%ID/Organ	%ID/g	%ID/Organ	%ID/Organ	%ID/g	%ID/Organ	%ID/Organ	%ID/g	%ID/Organ
0.08	0.6	0.1	6.5	0.04	1.8	3.8	2.1	0.3	3.061	0.20	5.4	3.141	1.4	1.8	2.885	0.2	0.5	3.061	0.3
0.25	1.8	0.1	7.7	0.05	2.0	4.2	2.3	0.3	3.162	0.21	6.9	3.997	1.7	1.7	2.624	0.2	0.6	3.162	0.28
0.50	3.5	0.0	3.3	0.02	1.6	3.3	1.9	0.2	2.700	0.18	6.0	3.466	1.5	1.1	1.674	0.1	0.5	2.700	0.2
0.75	5.3	0.1	5.7	0.04	1.9	4.0	2.2	0.4	4.442	0.29	13.5	7.828	3.4	1.1	1.724	0.1	0.8	4.442	0.4
1.00	7.1	0.1	7.5	0.05	2.4	5.0	2.8	0.4	4.725	0.31	11.2	6.509	2.8	1.1	1.688	0.1	0.8	4.725	0.4
1.50	10.6	0.1	8.1	0.05	1.9	3.9	2.2	0.3	3.507	0.23	10.7	6.199	2.7	0.7	1.090	0.1	0.6	3.507	0.3
2.00	14.2	0.1	7.4	0.05	2.3	4.8	2.7	0.5	6.034	0.40	11.1	6.482	2.8	1.4	2.197	0.1	1.1	6.034	0.5
3.00	21.3	0.1	5.3	0.03	2.4	5.0	2.8	0.4	4.488	0.30	12.5	7.272	3.2	1.3	1.988	0.12	0.8	4.488	0.4
4.00	28.3	0.1	9.0	0.06	2.7	5.7	3.2	0.5	5.352	0.35	2.1	1.237	0.5	0.8	1.223	0.1	0.9	5.352	0.5

Table S2. *Cont.*

Myocard			Kidneys			Liver			Lungs			Muscle			Ovaries			Pancreas		
Animal		Human	Animal		Human	Animal		Human	Animal		Human	Animal		Human	Animal		Human	Animal		Human
0.1583		316	0.3930		299	2.5203		1910	0.3025		1000	11.4216		28000	0.3539		8.71	0.1544		94.3
%ID/Organ	%ID/g	%ID/Organ	%ID/Organ	%ID/g	%ID/Organ	%ID/Organ	%ID/g	%ID/Organ	%ID/Organ	%ID/g	%ID/Organ	%ID/Organ	%ID/g	%ID/Organ	%ID/Organ	%ID/g	%ID/Organ	%ID/Organ	%ID/g	%ID/Organ
1.3	8.358	1.0	4.3	10.879	1.3	5.0	1.993	1.5	7.4	24.439	9.7	0.3	0.030	0.3	0.4	1.262	0.00	1.0	6.586	0.2
0.6	3.641	0.5	4.9	12.400	1.5	3.9	1.540	1.2	7.4	24.609	9.8	0.5	0.047	0.5	0.8	2.297	0.01	1.1	7.004	0.3
1.1	7.082	0.9	3.4	8.705	1.0	3.0	1.199	0.9	6.0	19.808	7.9	0.3	0.027	0.3	0.6	1.567	0.01	0.9	5.588	0.2
1.6	9.943	1.2	4.5	11.350	1.3	5.9	2.338	1.8	5.8	19.151	7.6	1.5	0.134	1.5	0.8	2.346	0.01	1.7	11.177	0.4
1.3	8.526	1.1	4.8	12.122	1.4	4.0	1.573	1.2	7.1	23.513	9.3	0.4	0.036	0.4	1.2	3.267	0.01	1.9	12.170	0.5
1.2	7.858	1.0	4.1	10.546	1.3	4.5	1.772	1.3	5.9	19.543	7.8	0.5	0.041	0.5	0.5	1.538	0.01	1.7	10.880	0.4
1.2	7.721	1.0	4.6	11.702	1.4	4.2	1.661	1.3	6.5	21.369	8.5	0.6	0.056	0.6	1.0	2.707	0.01	2.2	14.216	0.5
1.2	7.847	1.0	4.2	10.763	1.3	4.8	1.885	1.4	5.6	18.482	7.3	0.5	0.042	0.5	0.7	1.970	0.01	1.4	9.006	0.3
1.3	8.081	1.0	4.3	11.017	1.3	4.8	1.901	1.4	5.4	17.759	7.1	0.4	0.033	0.4	0.8	2.307	0.01	1.6	10.548	0.4

Table S2. *Cont.*

Red Marrow			Spleen			Thymus			ur. Bladder			Rem. of. Body		
Animal		Human	Animal		Human	Animal		Human	Animal		Human	Animal		Human
0.0384		1120	0.1126		183	0.0779		20.9	0.1153		47.6	10.4764		
%ID/Organ	%ID/g	%ID/Organ	%ID/Organ	%ID/g	%ID/Organ	%ID/Organ	%ID/g	%ID/Organ	%ID/Organ	%ID/g	%ID/Organ	%ID/Organ	%ID/g	%ID/Organ
0.1	2.233	1.0	0.3	3.026	0.2	0.2	2.233	0.02	0.2	1.569	0.0	69.4	6.627	80.4
0.1	2.682	1.2	0.7	6.539	0.5	1.3	16.392	0.14	0.5	4.243	0.1	66.7	6.366	79.7
0.1	2.522	1.1	0.6	5.512	0.4	0.3	3.705	0.03	1.0	8.315	0.2	73.4	7.003	83.1
0.1	2.304	1.0	0.7	6.535	0.5	0.2	2.463	0.02	1.0	8.300	0.2	58.5	5.582	77.9
0.2	4.204	1.9	0.7	6.647	0.5	0.2	2.222	0.02	3.4	29.533	0.6	58.8	5.613	76.6
0.2	3.986	1.8	0.5	4.067	0.3	0.2	3.154	0.03	1.8	15.829	0.3	64.5	6.159	79.8
0.1	3.787	1.7	0.8	7.095	0.5	0.4	4.581	0.04	0.9	7.992	0.2	60.9	5.817	77.7
0.2	4.310	1.9	1.0	8.944	0.6	0.2	2.154	0.02	1.6	13.862	0.3	61.3	5.848	78.5
0.1	3.742	1.7	0.8	7.055	0.5	0.2	3.030	0.03	3.5	30.348	0.6	69.5	6.632	80.9

Table S3. Mean %ID values of 3 female CD-1 mice after i.v. injection of 13.2 ± 3.0 MBq MBq (*S*)-(–)-[^{18}F]fluspidine followed by 105 min PET imaging.

Organ:		Brain			SI			Stomach			ULI		
Modell		Animal		Human	Animal		Human	Animal		Human	Animal		Human
Organmass (g)		0.4055		1420	1.8611		677	0.3397		158	0.1585		387
Fractions		%ID/Organ	%ID/g	%ID/Organ	%ID/Organ	%ID/g	%ID/Organ	%ID/Organ	%ID/g	%ID/Organ	%ID/Organ	%ID/g	%ID/Organ
$Time_{animal}$ (h)	$Time_{human}$ (h)												
0.00	0.0	2.9	4.2	2.5	7.0	4.6	1.3	2.4	3.4	0.2	1.1	2.8	0.5
0.08	0.6	2.6	3.8	2.3	10.5	6.4	1.9	1.8	2.4	0.2	1.2	3.1	0.5
0.17	1.2	2.5	3.8	2.3	12.1	7.6	2.2	1.7	2.4	0.2	1.2	2.9	0.5
0.25	1.8	2.5	3.7	2.3	13.5	8.7	2.5	1.8	2.5	0.2	1.2	3.2	0.5
0.33	2.4	2.5	3.8	2.3	15.8	10.5	3.0	1.8	2.5	0.2	1.3	3.2	0.5
0.50	3.5	2.5	3.7	2.2	16.9	11.2	3.3	1.9	2.6	0.2	1.2	3.1	0.5
0.75	5.3	2.4	3.6	2.2	16.9	10.9	3.2	1.9	2.6	0.2	1.3	3.2	0.5
1.00	7.1	2.4	3.6	2.2	17.5	11.2	3.2	1.9	2.6	0.2	1.4	3.3	0.5
1.25	8.8	2.4	3.6	2.1	17.3	11.1	3.2	2.1	2.8	0.2	1.5	3.5	0.6
1.50	10.6	2.4	3.5	2.1	15.9	10.3	3.0	2.2	3.0	0.2	1.5	3.5	0.6

Table S3. *Cont.*

Myocard			Kidneys			Liver			Lungs			Pancreas		
Animal		Human	Animal		Human	Animal		Human	Animal		Human	Animal		Human
0.2891		316	0.2923		299	1.4879		1910	0.7611		1000	0.0310		94
%ID/Organ	%ID/g	%ID/Organ	%ID/Organ	%ID/g	%ID/Organ	%ID/Organ	%ID/g	%ID/Organ	%ID/Organ	%ID/g	%ID/Organ	%ID/orgAn	%ID/g	%ID/Organ
1.8	5.7	0.8	7.3	23.4	3.0	17.2	11.5	9.3	6.9	7.7	3.2	0.0	0.0	0.0
0.9	2.9	0.4	7.6	24.2	3.1	16.1	10.7	8.7	4.2	4.7	2.0	0.0	0.0	0.0
0.8	2.7	0.4	7.6	24.2	3.1	14.5	9.7	7.9	3.8	4.3	1.8	0.0	0.0	0.0
0.8	2.7	0.4	7.6	24.3	3.1	13.4	8.9	7.3	3.8	4.2	1.8	0.0	0.0	0.0
0.8	2.4	0.3	7.4	23.8	3.0	12.3	8.2	6.6	3.5	4.0	1.7	0.0	0.0	0.0
0.7	2.2	0.3	6.6	21.2	2.7	11.4	7.6	6.2	3.2	3.5	1.5	0.0	0.0	0.0
0.6	2.0	0.3	4.8	15.6	2.0	10.0	6.7	5.4	2.9	3.3	1.4	0.0	0.0	0.0
0.6	1.9	0.3	3.8	12.4	1.6	9.3	6.2	5.0	2.8	3.1	1.3	0.0	0.0	0.0
0.6	1.9	0.3	3.5	11.2	1.4	9.0	6.0	4.9	2.7	3.0	1.3	0.0	0.0	0.0
0.6	2.0	0.3	2.9	9.2	1.2	8.9	5.9	4.8	2.8	3.2	1.3	0.0	0.0	0.0

Table S3. *Cont.*

Red Marrow			Spleen			Thyroid			ur. Bladder			Rem. of Body		
Animal		Human	Animal		Human	Animal		Human	Animal		Human	Animal		Human
1.2297		1120	0.0231		183	0.2049		20.7	0.3699		211	21.9614		
%ID/Organ	%ID/g	%ID/Organ	%ID/Organ	%ID/g	%ID/Organ	%ID/Organ	%ID/g	%ID/Organ	%ID/Organ	%ID/g	%ID/Organ	%ID/Organ	%ID/g	%ID/Organ
4.0	3.6	1.7	0.1	5.2	0.4	0.4	1.9	0.0	1.7	5.4	0.5	47.0	2.0	57.1
4.1	3.6	1.7	0.2	12.0	0.9	0.5	2.2	0.0	8.3	27.2	2.5	41.7	1.8	50.7
4.3	3.8	1.8	0.2	10.9	0.8	0.5	2.2	0.0	9.5	31.0	2.8	41.0	1.8	49.8
4.6	4.1	1.9	0.1	10.1	0.8	0.5	2.3	0.0	10.3	33.5	3.0	39.5	1.7	47.9
5.0	4.4	2.1	0.2	14.7	1.1	0.5	2.4	0.0	11.1	36.1	3.3	37.5	1.6	45.5
5.2	4.6	2.2	0.2	19.4	1.5	0.5	2.3	0.0	12.3	39.8	3.6	37.3	1.6	45.2
5.6	5.0	2.4	0.2	15.1	1.2	0.5	2.5	0.0	14.4	46.6	4.2	38.2	1.7	46.3
5.8	5.2	2.5	0.2	14.1	1.1	0.6	2.6	0.0	15.3	49.2	4.5	38.2	1.7	46.4
5.9	5.3	2.5	0.1	12.2	0.9	0.6	2.6	0.0	15.6	50.2	4.6	38.6	1.7	46.8
6.0	5.3	2.5	0.2	11.8	0.9	0.6	2.6	0.0	15.6	50.2	4.6	40.4	1.8	49.0

Table S4. Mean %ID values of 3 female CD-1 mice after i.v. injection of 12.6 ± 1.4 MBq MBq (*R*)-(+)-[^{18}F]fluspidine followed by 105 min PET imaging.

Organ:		Brain			SI			Stomach			ULI		
Modell		Animal		Human	Animal		Human	Animal		Human	Animal		Human
Organmass (g)		0.5032		1420	1.5725		677	0.5759		158	0.2160		387
Fractions		%ID/Organ	%ID/g	%ID/Organ	%ID/Organ	%ID/g	%ID/Organ	%ID/Organ	%ID/g	%ID/Organ	%ID/Organ	%ID/g	%ID/Organ
$Time_{animal}$ (h)	$Time_{human}$ (h)												
0.00	0.0	3.1	6.3	3.7	7.7	5.0	1.4	2.3	4.2	0.3	1.1	4.7	0.8
0.08	0.6	3.2	6.4	3.8	10.4	6.7	1.9	3.1	5.6	0.4	1.7	6.7	1.1
0.17	1.2	2.6	5.2	3.1	12.3	7.9	2.2	3.1	5.6	0.4	1.5	6.3	1.0
0.25	1.8	2.2	4.4	2.6	15.4	9.8	2.8	3.0	5.4	0.4	1.4	6.4	1.0
0.33	2.4	1.8	3.7	2.2	17.9	11.4	3.2	2.9	5.3	0.3	1.7	8.2	1.3
0.50	3.5	1.5	3.0	1.8	19.3	12.4	3.5	3.2	5.7	0.4	2.0	10.2	1.7
0.75	5.3	1.3	2.6	1.6	19.6	12.6	3.6	3.1	5.6	0.4	2.3	12.2	2.0
1.00	7.1	1.2	2.4	1.4	19.1	12.3	3.5	2.8	5.0	0.3	2.0	9.6	1.6
1.25	8.8	1.2	2.3	1.4	18.9	12.1	3.4	2.9	5.2	0.3	2.7	12.0	2.0
1.50	10.6	1.2	2.4	1.4	19.3	12.4	3.5	2.7	5.1	0.3	3.1	13.3	2.2

Table S4. *Cont.*

**Myocard**			**Kidneys**			**Liver**			**Lungs**			**Pancreas**		
**Animal**		**Human**	**Animal**		**Human**	**Animal**		**Human**	**Animal**		**Human**	**Animal**		**Human**
**0.2911**		**316**	**0.3490**		**299**	**1.3634**		**1910**	**0.7892**		**1000**	**0.0154**		**94**
**%ID/Organ**	**%ID/g**	**%ID/Organ**	**%ID/Organ**	**%ID/g**	**%ID/Organ**	**%ID/Organ**	**%ID/g**	**%ID/Organ**	**%ID/Organ**	**%ID/g**	**%ID/Organ**	**%ID/Organ**	**%ID/g**	**%ID/Organ**
1.6	5.4	0.7	4.6	13.0	1.6	12.4	9.0	7.2	5.1	6.5	2.7	0.1	7.4	0.3
1.1	3.6	0.5	4.9	13.8	1.7	17.0	12.3	9.7	3.6	4.4	1.8	0.2	10.3	0.4
0.9	2.9	0.4	4.2	11.8	1.5	14.4	10.5	8.3	2.9	3.5	1.4	0.2	10.3	0.4
0.8	2.6	0.3	3.7	10.4	1.3	12.5	9.0	7.2	2.6	3.0	1.3	0.2	10.5	0.4
0.7	2.2	0.3	3.3	9.4	1.2	10.4	7.5	6.0	2.2	2.6	1.1	0.2	10.2	0.4
0.6	1.9	0.2	2.9	8.1	1.0	8.2	5.9	4.7	1.8	2.1	0.9	0.1	8.1	0.3
0.5	1.6	0.2	2.6	7.3	0.9	7.0	5.1	4.0	1.7	2.0	0.8	0.1	7.2	0.3
0.6	1.9	0.3	2.2	6.1	0.8	6.5	4.7	3.8	1.9	2.3	1.0	0.1	5.1	0.2
0.6	1.9	0.2	2.1	5.8	0.7	6.5	4.7	3.7	1.9	2.3	0.9	0.1	5.4	0.2
0.6	1.9	0.2	2.0	5.5	0.7	6.6	4.8	3.8	1.9	2.3	1.0	0.1	4.9	0.2

Table S4. *Cont.*

**Red Marrow**			**Spleen**			**Thyroid**			**ur. Bladder**			**Rem. of. Body**		
**Animal**		**Human**	**Animal**		**Human**	**Animal**		**Human**	**Animal**		**Human**	**Animal**		**Human**
**1.4825**		**1120**	**0.0327**		**183**	**0.2357**		**20.7**	**0.1324**		**211**	**23.2667**		
**%ID/Organ**	**%ID/g**	**%ID/Organ**	**%ID/Organ**	**%ID/g**	**%ID/Organ**	**%ID/Organ**	**%ID/g**	**%ID/Organ**	**%ID/Organ**	**%ID/g**	**%ID/Organ**	**%ID/Organ**	**%ID/g**	**%ID/Organ**
5.5	3.7	1.7	0.2	6.1	0.5	0.5	2.2	0.0	0.1	1.0	0.1	55.3	2.4	65.3
7.3	4.9	2.3	0.3	11.3	0.9	0.8	3.3	0.0	1.5	10.8	0.9	44.8	1.9	52.7
7.7	5.2	2.4	0.5	14.0	1.1	0.9	3.5	0.0	3.1	24.2	2.1	45.7	1.9	53.7
8.3	5.6	2.6	0.5	15.8	1.2	0.9	3.6	0.0	5.6	43.6	3.8	42.8	1.8	50.3
9.3	6.3	2.9	0.6	16.2	1.2	0.9	3.6	0.0	7.3	57.8	5.1	40.7	1.7	47.7
10.8	7.3	3.4	0.5	15.4	1.2	0.9	3.7	0.0	9.5	75.4	6.6	38.3	1.6	44.9
11.9	8.1	3.8	0.5	14.8	1.1	0.9	3.6	0.0	10.5	82.5	7.3	37.6	1.6	44.1
10.1	7.0	3.3	0.4	12.1	0.9	0.7	3.0	0.0	10.2	78.9	6.9	41.7	1.8	49.0
10.8	7.4	3.5	0.4	10.3	0.8	0.7	3.0	0.0	10.9	84.3	7.4	40.0	1.7	47.0
11.0	7.7	3.6	0.3	9.0	0.7	0.7	2.9	0.0	9.5	73.8	6.5	40.5	1.7	47.6

Table S5. Mean %ID values of 4 volunteers after i.v. injection of 255 ± 9 MBq (*S*)-(−)-[^{18}F]fluspidine followed by 6 h PET imaging.

Organ:	Brain	Gallbladder	LLI	SI	Stomach	ULI	Myocardium	Kidneys
**Organmass (g)**	**1304**	**18**	**110**	**1619**	**502**	**82**	**162**	**278**
**Time (h)**	**%ID**	**%ID**	**%ID**	**%ID**	**%ID**	**%ID**	**%ID**	**%ID**
0.00	1.8	0.2	0.4	8.2	2.6	0.4	1.4	3.4
0.25	8.6	0.3	0.4	8.5	3.1	0.5	1.2	2.2
0.50	8.3	0.4	0.5	8.1	3.2	0.4	1.0	1.7
0.75	7.5	0.4	0.5	7.8	3.1	0.4	0.9	1.4
1.00	6.7	0.5	0.4	7.6	3.0	0.4	0.7	1.3
1.33	5.6	0.8	0.5	7.6	2.8	0.4	0.6	1.3
1.67	4.8	1.0	0.5	8.0	2.7	0.4	0.5	1.2
3.00	3.4	1.3	0.3	9.8	1.4	0.8	0.3	1.0
4.50	2.3	1.8	0.2	11.8	1.2	0.4	0.3	0.8
6.00	1.8	1.4	0.2	10.9	1.2	1.4	0.3	1.0

Table S5. *Cont.*

Liver	Lung	Red Marrow	Spleen	Testes	Thyroid	ur. Bladder	Rem. of Body
**1634**	**1925**	**3655**	**75**	**56**	**21**	**564**	**38054.0**
%ID	%ID	%ID	%ID	%ID	%ID	%ID	%ID
11.6	24.0	6.6	2.1	0.0	0.1	0.2	38.3
18.8	8.8	8.2	1.5	0.0	0.0	0.4	37.9
22.6	6.0	8.4	1.0	0.0	0.0	0.7	38.6
25.2	5.0	8.5	0.8	0.0	0.0	1.1	38.5
26.8	4.4	8.4	0.6	0.0	0.0	1.9	38.5
27.4	4.2	8.5	0.5	0.0	0.0	2.9	38.0
27.7	3.9	8.4	0.5	0.0	0.0	4.0	37.4
24.5	3.4	8.0	0.3	0.0	0.0	3.6	39.5
20.1	2.8	7.6	0.3	0.0	0.0	3.2	34.2
16.1	2.7	7.0	0.3	0.0	0.0	3.0	32.0
